# Utilization of wild desert plant extracts for the *in vitro* control of gastrointestinal nematodes in cattle

**DOI:** 10.5455/javar.2025.l917

**Published:** 2025-06-02

**Authors:** Raquel Olivas-Salazar, Ana Verónica Charles-Rodríguez, Fidel Maximiano Peña Ramos, Joel Ventura-Ríos, Fernando Ruiz Zárate, Roberto González Garduño

**Affiliations:** 1 Departamento de Producción Animal, Universidad Autónoma Agraria Antonio Narro, Saltillo, México; 2 Departamento de Ciencia y Tecnología de Alimentos, Universidad Autónoma Agraria Antonio Narro, Saltillo, México; 3 Departamento de Ciencias del suelo, Universidad Autónoma Agraria Antonio Narro, Saltillo, México; 4 Universidad Autónoma Chapingo, Unidad Regional Universitaria Sursureste, Teapa, México

**Keywords:** Egg hatching, infective larvae, prasite control, probit

## Abstract

**Objective:**

This study aimed to evaluate the effects of plant extracts from desert species on egg-hatching inhibition (EHI) and larval mortality of gastrointestinal nematodes (GINs) in cattle under *in vitro* conditions.

**Materials and Methods:**

Hydro-alcoholic extracts of tasajillo [*Cylindropuntia leptocaulis* (DC) F.M. Kunth], coyonoxtle [*Cylindropuntia imbricata* (Haw) F.M. Kunth], mariola (*Parthenium incanum* Kunth), and mesquite [*Neltuma juliflora* (Sw.) Raf.] were tested. GIN eggs and infective larvae were exposed to the plant extracts in decreasing doses, and the median lethal dose_50_ (LD_50_) and maximum lethal dose_99_ (LD_99_) were calculated using the SAS PROBIT procedure.

**Results:**

The *C*. *imbricata* extract demonstrated the highest EHI at a small LD_50_ (2.31 mg/ml) and achieved 100% larval mortality at a 5.8 mg/ml concentration. The *P*. *incanum* extract showed the highest larval mortality at the LD_99_ (6.50 mg/ml), although *N*. *juliflora* had the lowest LD_50_. However, the *N*. *juliflora* pod extract was the least effective overall. These findings indicate that *C. imbricata* was the most effective extract for inhibiting egg hatching, while *P. incanum* was the most effective for promoting larval mortality under *in vitro* conditions.

**Conclusion:**

The small doses used against eggs and larvae of nematode parasites suggest that wild desert plants could provide a viable and ecological alternative for the *in vitro* control of GINs in cattle.

## Introduction

Cattle production in pasture-based systems faces several challenges that impact farm productivity and sustainability. These challenges include providing adequate feed for animals, adapting to increasingly extreme climate variations (cold and hot temperatures) [1,2], and controlling diseases, particularly those caused by endoparasites [3,4]. Gastrointestinal nematode (GIN) infections are among the most significant threats, leading to weight loss, anemia, decreased milk and meat production, general weakness, and in severe cases, mortality [5]. Endoparasites not only compromise animal health and welfare but also reduce the sector’s profitability, causing substantial economic losses for farmers [6,7].

In recent years, the growing threat of anthelmintic resistance has compounded the challenges of controlling GINs. This resistance reduces or eliminates the effectiveness of chemical products against GINs [8] because parasite populations develop genetic adaptations that enable them to survive and multiply despite treatment with combinations of anthelmintics. As a result, infection burdens increase, necessitating more expensive treatments, which can also contaminate the environment and negatively impact soil microorganisms [9,10]. To address this issue, various practices have been implemented to control parasitic diseases, including proper grazing management [11], the use of animals resistant to parasitic nematodes [12], and selective deworming [13], among other methods. The most promising alternative is the use of plant extracts as anthelmintic agents [14]. The extracts contain bioactive compounds such as tannins, saponins, flavonoids, terpenes, and phenolic derivatives (e.g., eugenol), which have shown ovicidal and larvicidal effects both *in vitro* and *in vivo* [15–18] and activity against a wide range of microorganisms [19,20]. Plant extracts offer an attractive option for controlling GINs because they effectively target GIN eggs and larvae while providing an ecological and economic alternative. Unlike synthetic anthelmintics, plant extracts do not affect the health of the soil, making them a sustainable long-term solution for managing parasitic infections in livestock systems [21]. In the arid and semi-arid regions of northern Mexico, where free-range animal populations are substantial, GINs pose a significant problem. In this context, some desert plants have demonstrated effective larvicidal and ovicidal properties for GIN control [22]. It is important to evaluate many wild plants that produce secondary metabolites that affect GINs and that can be used in the control of these parasites.

The hypothesis proposed was that because of the presence of their chemical components, some desert plants exhibit ovicidal and larvicidal effects on bovine GINs under *in vitro* conditions. The objective of this study was to determine the *in vitro* effect of wild desert plant extracts on the inhibition of egg hatching and larval mortality of bovine GINs using the species tasajillo (*Cylindropuntia leptocaulis*), coyonoxtle (*Cylindropuntia imbricata*), mariola (*Parthenium incanum*), and mesquite (*Neltuma juliflora*).

## Materials and Methods

### Ethical approval

Ethical approvals were obtained after project 38111-4252020012101 was approved by the programming and evaluation sub-directorate of the research department of the Antonio Narro Autonomous Agrarian University and followed the Mexican Official Standard NOM-051-ZOO-1995, on humane treatment in the mobilization of animals and NOM-062-ZOO-1999 on technical specifications for the production, care and use of laboratory animals. This *in vitro* study was conducted in the animal health laboratory of the South-Southeast Regional University Unit of the Autonomous University of Chapingo, located in San José Puyacatengo, Municipality of Teapa, Tabasco, Mexico. The study site is situated at an altitude of 60 m. above sea level, with coordinates 17° 34' 30" N latitude and 92° 56' 15" W longitude. The region’s climate is classified as equatorial rainforest, fully humid [23], with an average annual temperature of 25.8°C and annual precipitation of 3,975.5 mm.

### Collection of plant species

The plants selected for this study included species believed to have anthelmintic properties, such as those from the genera *Cylindropuntia* [24], *Parthenium* [25], and *Neltuma* [26]. The collected plant species were verified against specimens in the repository of the National Herbarium of Mexico, with the following catalog numbers [27]: stem of tasajillo (*C. leptocaulis* (DC) FM Knuth, 1246285), stem of coyonoxtle (*C. imbricata* (Haw) DC, 900988), whole plant of mariola (*P. incanum* Kunth, 475512), and pod of mesquite (*N. juliflora* (Sw) DC, 1366067). These plants were collected in the Agua Nueva ejido, Municipality of Saltillo, Coahuila, located at coordinates 25° 11' 16'' N and 101° 04' 55'' W.

### Preparation of extracts

The hydroalcoholic extracts were prepared using the conventional method described in a recent study [28]. Briefly, 125 ml of ethanol (50:50 ratio with water) was used as a solvent for 11.5 gm of ground vegetative material, which had been passed through a 20-mesh sieve (0.84 mm). The mixture was stirred at 150 rpm for 24 h at 25°C and then filtered through Whatman No. 1 paper, yielding 120 ml of extract. Table 1 presents the bromatological values of each plant species used to produce the extracts.

### Nematode egg collection

Fecal samples were collected directly from the rectum of five naturally infected calves grazing on *Urochloa decumbens* grass. The calves were 5/8 Holstein × 3/8 Zebu crossbreds, 4 months old, and weighed an average of 95 kg. Their diet consisted of grazing supplemented with 1 kg of feed prepared at the experimental farm, containing 16% crude protein and 2.8 Mcal of metabolizable energy.

**Table 1.** Bromatological analysis (Weende analysis) of the plant species used for extract preparation.

Extract	Scientific name	Moisture	Fat	Protein	Ash	Fiber	NFE
Coyonoxtle	*Cylindropuntia imbricata*	6.54	2.06	0.07	32.24	11.98	48.28
Mariola	*Parthenium incanum*	5.62	7.10	4.99	11.19	25.79	50.93
Mesquite	*Prosopis juliflora*	7.31	6.82	6.86	5.62	22.89	57.81
Tasajillo	*Cylindropuntia leptocaulis*	26.12	2.89	1.84	9.21	15.20	70.86

NFE = nitrogen-free extract.

A pooled fecal sample was prepared from calves exceeding 400 eggs per gm to obtain nematode eggs. The feces were mixed with a saturated saline solution (density = 1.20) at a ratio of 2 gm of feces per 14 ml of saline solution. The mixture was transferred to 50-ml conical tubes (Falcon^®^) and centrifuged at 2,500 rpm for 5 min. The supernatant was then washed with running water using a 100-mesh sieve (0.088 mm; Mont-inox) paired with a 400-mesh sieve (0.037 mm; Mont-inox) at the bottom. Eggs retained on the bottom sieve were collected and transferred to 15-ml tubes, where 6 ml of saturated sucrose solution (density = 1.28) was added as the flotation liquid. The mixture was centrifuged again at 2,500 rpm for 5 min. The ring formed in the middle of the tube was recovered using a Pasteur pipette and washed with running water through the 400-mesh sieve. The eggs recovered from this sieve (approximately 10 ml) were transferred to a 15-ml conical tube. The egg concentration was determined by counting 10 aliquots of 10 µl and adjusting the volume to achieve a final concentration of 100 eggs per 100 µl.

### Infective larvae collection

The infective larvae used in the bioassays were obtained from coprocultures prepared with a portion of the collected feces, and incubated for 8 days at room temperature. The feces were mixed with foam rubber in a container covered with gauze. The larvae were then cleaned using a Baermann apparatus, which removed impurities and concentrated the live larvae into a test tube. The identification of the larvae was carried out by morphology based on the presence of the sheath, shape of tail, and size of infective larvae [29].

### Egg hatching test and larval mortality

The *in vitro* test, which included egg hatching and larval mortality assessments, was conducted using plant extracts alongside their respective controls. The negative control consisted of distilled water and methanol, while the positive control used a commercial anthelmintic (fenbendazole) (PANACUR^®^; MSD Animal Health) due to its ovicidal capacity and because this product had not been used on the herd. Larvae were categorized based on the following criteria: dead larvae were identified as straight and immobile, while live larvae were coiled or exhibited movement [30]. The larval counts were made without differentiating species and reported as GINs.

The evaluations were performed in 96-well polystyrene plates. Each well contained third-stage larvae (L_3_) or 100 eggs suspended in 100 µl of water, to which 100 µl of plant extracts diluted in phosphate-buffered saline with 1% Tween 20 were added. From the first well, 100 µl of each treatment (23 mg/ml) was taken, and serial dilutions were prepared to achieve the following concentrations: 11.5, 5.75, 2.87, 1.44, and 0.72 mg/ml. Wells containing distilled water with methanol (because the extracts were in ethanol) at 12.5%, 6.25%, 3.125%, 1.56%, and 0.78% served as the negative control. After the extracts were added, the plate was covered with wax paper to prevent evaporation and incubated at 25°C for 24 h.

The assessment of egg hatching inhibition (EHI) and larval mortality was performed 24 h after the extracts were applied. Five 20-µl aliquots were taken from each well, and the unhatched eggs, first-stage larvae, and live or dead third-stage larvae (L_3_) were counted for each treatment, as well as for the positive and negative controls.

The percentage of EHI was calculated using the following formula [18]:

$\mathrm{EHI}\mathrm{(\%)}\;=\;\frac{\mathrm{number}\;\mathrm{of}\;\mathrm{unhatched}\;\mathrm{eggs}}{\mathrm{number}\;\mathrm{of}\;\mathrm{unhatched}\;\mathrm{eggs}+\mathrm{number}\;\mathrm{of}\;L1\;\mathrm{larvae}}\times 100$

The percentage of larval mortality (LM) was calculated as follows [18]:

$\mathrm{LM}\mathrm{(\%)}\;=\;\frac{\mathrm{number}\;\mathrm{of}\;\mathrm{dead}\;\inf\mathrm{ective}\;\mathrm{larvae}}{\mathrm{total}\;\mathrm{number}\;\mathrm{of}\;\mathrm{larvae}\;\mathrm{counted}}\times 100$

The data obtained were analyzed to obtain the lethal dose (LD_50_) for each extract using PROBIT analysis in the SAS program [31] based on the following model:

Pr(Response) = C + (1 − C)F(x‘β) = C + (1 − C)Φ(b_0_ + b_1_ × log_10_(Dose))

where β is a vector of estimated parameters. F is the cumulative distribution function (normal). x is a vector of explanatory variables. Pr is the probability of a response. C is the natural response rate (proportion of individuals responding to dose zero).

Additionally, a statistical analysis was performed using a completely randomized design in a one-way model to determine differences between the concentrations. Mean comparisons were carried out using Tukey’s test.

## Results

Table 2 presents the results of the PROBIT analysis for the GIN EHI test and the larval mortality test, where eggs and larvae were exposed to extracts from the four desert plants. A dose-dependent response was observed in all cases, as indicated by the significant slope values. Additionally, the natural mortality rate of the larvae was <7% in all cases. In the EHI test, a dose-dependent response was also evident.

### EHI test

In the EHI test, *C*. *imbricata* extract demonstrated the lowest LD_50_ and LD_99_ values, with the narrowest confidence limits, making it the most effective product against GIN eggs. Extracts from *C*. *leptocaulis*, *P*. *incanum*, and *N*. *juliflora* showed similar LD_50_ and LD_99_ values, although they were higher than those of *C*. *imbricata* (Table 3).

**Table 2.** Parameters derived from PROBIT analysis of plant extracts from desert plants.

Plant extract	Scientific name	Kind	C	β_0_	SE	β_1_	SE
Coyonoxtle	*Cylindropuntia imbricata*	Larvae	0.005	–4.52**	0.94	6.70**	1.39
		Eggs	0.01	–2.94**	0.47	8.07**	1.09
Mariola	*Parthenium incanum*	Larvae	0.038	–5.51**	0.64	9.64**	1.19
		Eggs	0.02	–2.42**	0.48	4..12**	0.77
Mezquite	*Prosopis juliflora*	Larvae	0.002	–0.77^ns^	0.51	1.58**	0.58
		Eggs	0.03	–2.18**	0.40	3.88**	0.63
Tasajillo	*Cylindropuntia leptocaulis*	Larvae	0.076	–4.92**	1.13	7.21**	1.46
		Eggs	0.02	–2.25**	0.29	3.94**	0.46

β_0_ = intercept; β_1_ = slope; C = natural mortality rate (1-C = survival rate).

*Significant (*p* < 0.05).

**Highly significant (*p* < 0.01).

^ns^ Not significant.

**Table 3.** Median and maximum lethal doses (LD_50_ and LD_99_) of egg hatching inhibition of bovine gastrointestinal nematodes.

Extract	LD_50_ (mg/ml)	95% fiducial limits		LD_99_ (mg/ml)	95% fiducial limits		Chi^a^
		Lower	Upper		Lower	Upper	
*Cylindropuntia leptocaulis*	3.7	3.3	4.2	14.4	10.9	22.0	72.5
*Cylindropuntia imbricata*	2.3	2.1	2.5	4.5	3.8	5.9	54.6
*Parthenium incanum*	3.9	2.6	5.6	14.1	8.2	100.9	28.5
*Neltuma juliflora*	3.6	2.1	5.5	14.4	7.9	328.6	37.7

^a^Chi-square value, significant at p < 0.05 level.

**Table 4.** Egg hatching inhibition (%) of bovine gastrointestinal nematodes with different desert plant extracts.

Plant extract	Doses (mg/ml)			
	5.8	2.9	1.4	0.7
Cylindropuntia leptocaulis	73.8 ± 26.2^ab^	41.8 ± 5.8^bc^	2.6 ± 2.6^c^	1.4 ± 1.4^c^
Cylindropuntia imbricata	100ª	76.6 ± 6.6^ab^	6.1 ± 0.9^c^	2.6 ± 2.6^c^
Parthenium incanum	72.5 ± 27.5^ab^	39.8 ± 38.1^bc^	4.9 ± 4.9^c^	0.4 ± 0.4^c^
Neltuma juliflora	63.7 ± 36.3^ab^	36.9 ± 36.5^bc^	-	-

Different literals demonstrate statistically significant differences (*p* < 0.05).

**Figure 1.**
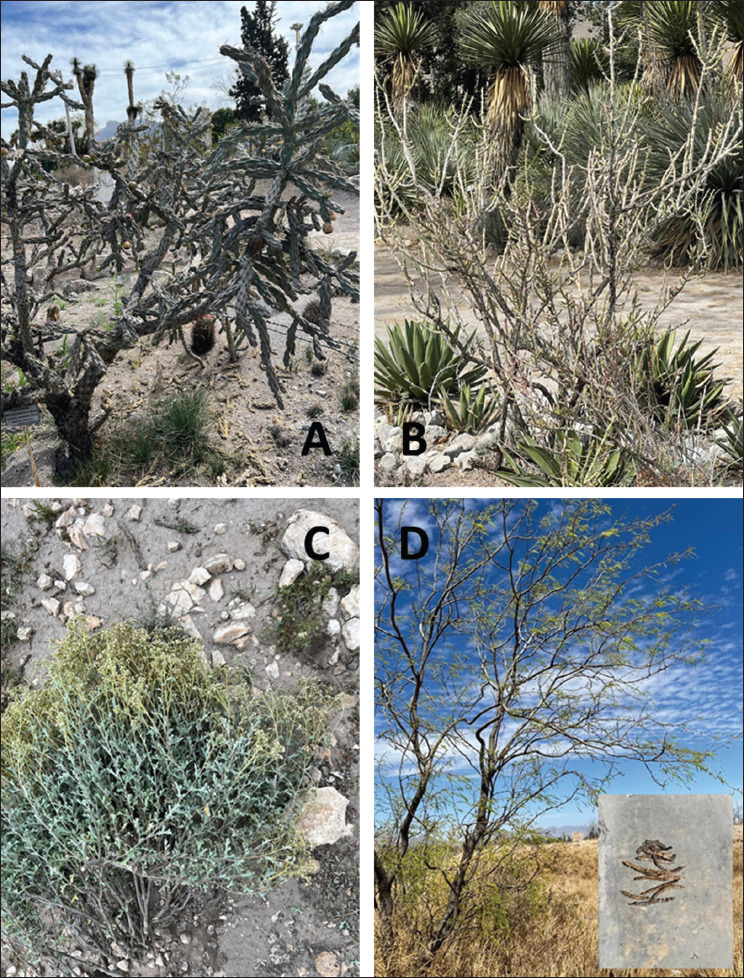
Wild desert plants used to obtain extracts for the control of gastrointestinal nematodes in cattle. (A) Coyonoxtle [*Cylindropuntia imbricata* (Haw) F.M. Kunth], (B) Tasajillo [*Cylindropuntia leptocaulis* (DC) F.M. Kunth], (C) Mariola (*Parthenium incanum* Kunth), (D) Mesquite [*Neltuma juliflora* (Sw.) Raf.].

When analyzing the dose response in a one-way model, it was observed that doses of >5.8 mg/ml caused mortality rates of >63% for *N*. *juliflora* and >72% for *C*. *leptocaulis* and *P*. *incanum*. *Cylindropuntia imbricata*, on the other hand, achieved a mortality rate of 100% at this dose (Table 4; Fig. 1).

**Figure 2.**
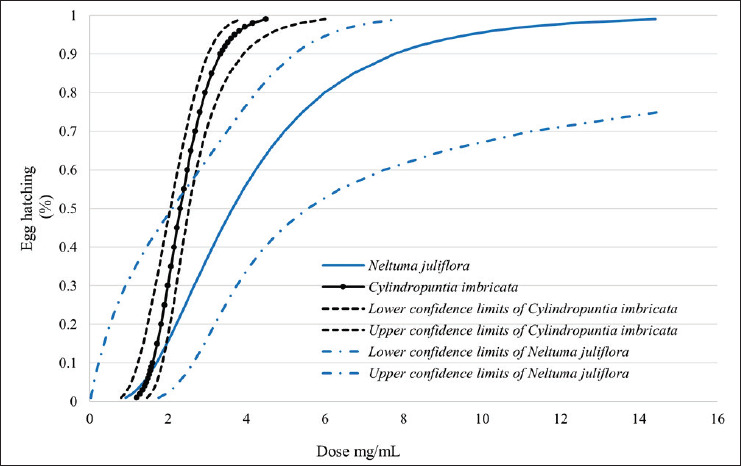
PROBIT curves derived from the analysis of *Neltuma juliflora* and *Cylindropuntia imbricata* on the hatching inhibition of bovine gastrointestinal nematode eggs. *N*. *juliflora* LD_50_ = 3.6 mg/ml, *C*. *Imbricata* LD_50_ = 2.3 mg/ml.

The confidence intervals for the *C. imbricata* extract were very narrow, indicating that both the LD_50_ and LD_99_ were highly effective in inhibiting GIN egg hatching. By contrast, *N. juliflora* extracts had a slightly higher LD_50_ than *C. imbricata*, but their LD_99_ was considerably further away, suggesting that *N*. *juliflora* is a less effective product (Fig. 2).

## Larval mortality assay

In the fecal cultures, the nematode composition was 70% *Cooperia* sp., 20% *Oesophagostomum* sp., 5% *Haemonchus* sp., and 5% *Strongyloides* sp., morphologically identified. However, the results are only reported as GIN.

Among the plant extracts, *N*. *juliflora* exhibited the lowest LD_50_. However, the concentration required to achieve 99% larval mortality (LD_99_) was higher than that of the other extracts, at 91.49 mg/ml. This indicates that *N*. *juliflora* is less efficient than *C*. *leptocaulis*, *C*. *imbricata*, and *P*. *incanum*, which showed similar values for both LD_50_ and LD_99_ (Table 5).

**Figure 3.**
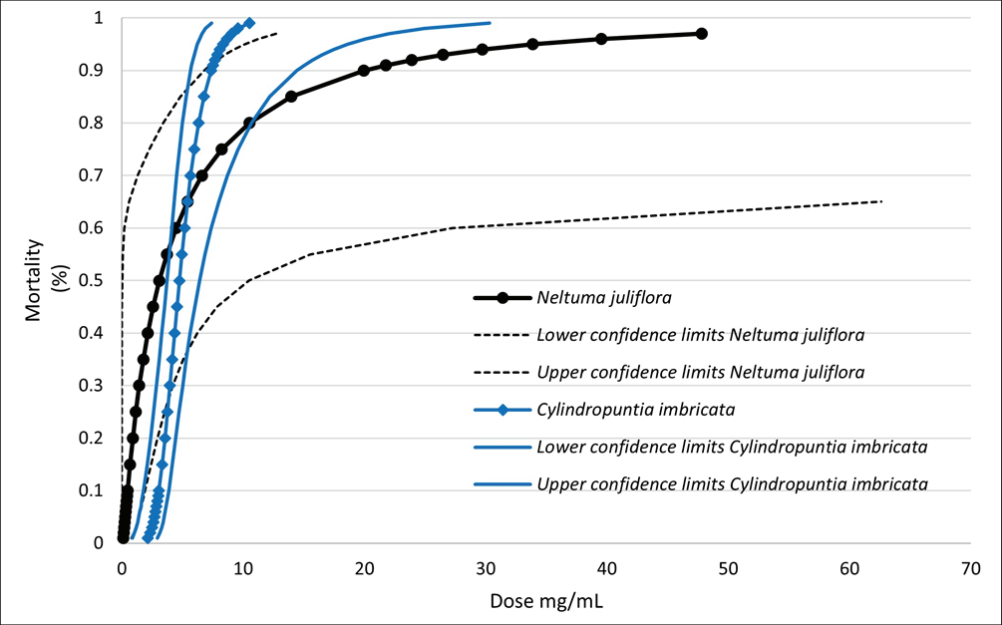
Mortality rate of gastrointestinal nematode larvae according to the doses of *Neltuma juliflora* and *Cylindropuntia imbricata* extracts, including the lower and upper 95% confidence limits. *N*. *juliflora* LD_50_ = 3.08 mg/ml, *C*. *Imbricata* LD_50_ = 4.73 mg/ml.

Although *N*. *juliflora* had the lowest LD_50_, its 99% effectiveness required a very high concentration, and the confidence limits increased significantly after a concentration of 50 mg/ml (Fig. 3). By contrast, although *C*. *imbricata* also showed high confidence limits after LD_80_, it can be concluded that *C*. *imbricata* performed better in reducing larval motility of bovine GINs.

**Figure 4.**
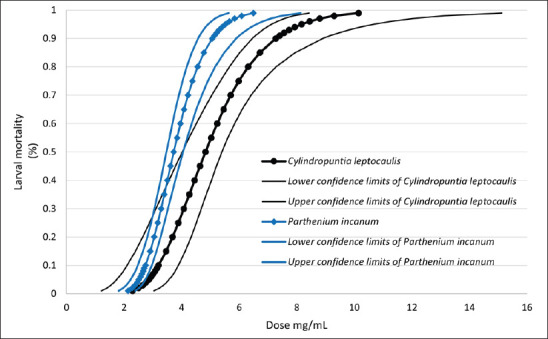
Mortality rate of gastrointestinal nematode larvae according to the doses of *Cylindropuntia leptocaulis* and *Parthenium incanum* extracts, including the lower and upper 95% confidence limits. *C*. *leptocaulis* LD_50_ = 4.83 mg/ml, *P*. *incanum* LD_50_ = 3.73 mg/ml.

*Cylindropuntia leptocaulis* and *P. incanum* exhibited similar behavior. Although *C. leptocaulis* had the highest upper confidence interval, the similarity of the figures indicates comparable effectiveness against GIN larvae (Fig. 4).

**Table 5.** Median and maximum lethal doses (LD_50_ and LD_99_) on larval mortality of gastrointestinal nematodes.

Plant extract	LD_50_	95% fiducial limits		LD_99_	95% fiducial limits		
	(mg/ml)	Lower	Upper	(mg/ml)	Lower	Upper	Chi^a^
*Cylindropuntia leptocaulis*	4.83	4.02	5.40	10.15	8.43	15.12	24**
*Cylindropuntia imbricata*	4.73	3.70	6.39	10.53	7.38	30.32	23**
*Parthenium incanum*	3.73	3.47	4.05	6.50	5.63	8.14	65.6**
*Neltuma juliflora*	3.08	0.02	10.49	91.49	19.12	>152	7.6**

**Highly significant (*p* < 0.01).

## Discussion

In Mexico, as in many other countries, various alternatives have been explored to address the issue of anthelmintic resistance in sheep, goats [32,33], and cattle. These alternatives include improved feeding strategies to enhance immunity, the application of the FafaMalanChart technique to deworm only animals in need, and genetic selection of hosts to promote resistance [34]. Pasture management [4], the search for vaccines, and the biological control of nematode parasites of cattle have become important [35]. However, with the reduced effectiveness of anthelmintic products, which has been widely documented for several species of bovine nematodes, which has caused an increase in costs both for treatment and for the effect of low weight gain in animals and subclinical health effects [7], it has become essential to integrate multiple control methods to maintain productive animals [36].

The use of secondary metabolites from plants has been a natural alternative option explored by numerous researchers for the control of GINs. These metabolites play a crucial role in plant defense against herbivores, viruses, bacteria, fungi, and parasites, sparking renewed interest in their application in veterinary medicine [37]. Among these metabolites, polyphenolic compounds, such as tannins present in plant extracts, have been evaluated for their effectiveness in controlling nematodes in cattle and sheep [38]. These compounds can bind to proteins in the host animal’s intestinal tract or to glycoproteins in the parasite’s cuticle, ultimately causing the nematode’s death [39,40]. Additionally, these compounds may directly affect the viability of pre-parasitic stages of helminths, positioning their use as a promising alternative to reduce chemical anthelmintics, promote sustainability, and decrease environmental pollution [35]. Other secondary metabolites, including essential oils, flavonoids, and terpenoids, have also been suggested to possess anthelmintic properties [41–43].

Plant extracts have long been used as a traditional alternative for controlling gastrointestinal parasitosis. Recently, they have regained attention because of their potential to mitigate the rise of anthelmintic resistance, which has developed over time as a result of excessive anthelmintic use in sheep, goat, and cattle production worldwide [37,44].

In this study, both egg hatching and the larval mortality of GIN in cattle were concentration-dependent, with significance evident in the slope of the regression curve. A similar trend was reported in another study [45], where higher concentrations of *Pluchea sericea* and *Artemisia tridentata* extracts per unit of solvent resulted in increased mortality.

In the case of *C. imbricata*, 100% inhibition of egg hatching was achieved by applying 5.8 mg of extract per ml of solvent. Regarding larval mortality, the product with the lowest LD_99_ was *P*. *incanum*, with a value of 6.5 mg/ml. No previous studies were found on the application of *C. imbricata* and *C. leptocaulis* against GINs, making this study the first to evaluate their *in vitro* application against eggs and larvae of GINs in cattle.

Although a review in Cactus indicates the presence of alkaloids, terpenoids, steroids, isocoumarins, chromones, phenolic saturated and unsaturated fatty acids, and aromatic compounds in the *Opuntia* and *Cylindropuntia* genus, its application has primarily been limited to its antifungal properties but suggests a biotechnological potential [46]. The dose dependence observed with *C*. *imbricata* was also reported in a study [22], which showed that increasing the concentration of *Opuntia ficus* extract was associated with a higher percentage of inhibition in the egg hatching of *Haemonchus contortus* from sheep.

In the case of *P*. *incanum*, this plant has been used in traditional Mexican medicine to treat gastrointestinal diseases [47] and has been evaluated for its effects against the hemoflagellate protozoan *Trypanosoma cruzi*. Observing the presence of secondary metabolites such as alkaloids, flavonoids, sesquiterpene lactones, sterols, triterpenes, and tannins, which have been the main components evaluated in the *in vitro* control of GINs [25,26]. However, no reports are available on its use against GINs in ruminants.

Although the *N*. *juliflora* pod exhibited the lowest LD_50_, it performed poorly at the LD_99_, requiring a very high concentration (91.49 mg/ml). However, many *Prosopis* species, including *Prosopis juliflora*, have been used in human medicine to treat various conditions, such as respiratory problems, pain, diabetes, diarrhea, liver infections, malaria, rheumatism, skin inflammations, stomach pain, and others [48]. This plant is widely available in northern Mexico, making it easy to obtain. Its chemical composition includes alkaloids, tannins, saponins, flavonoids, and triterpenes, which have been utilized to control GINs in goats [26].

Additionally, its effects have been evaluated in studies on EHI of GINs in sheep [49] and specifically on *H*. *contortus* [50]. These findings suggest that *N*. *juliflora* could be of interest to producers, especially with further studies on its effectiveness. It is also common practice in the field to supplement grazing ruminants with *N*. *juliflora* pods, which could support its potential use as a probiotic.

The challenges of using extracts involve the multiple evaluation of the extracts and components, due to changes in the concentration of secondary compounds due to environmental factors, knowing the stability of the components, and the cost of obtaining an extract, as well as determining the appropriate doses in *in vivo* studies. Although a dose-dependent response was obtained both in nematode egg hatching and in larval mortality, it is necessary to corroborate the ovicidal or larvicidal response in *in vivo* studies since some components undergo structural changes during passage through the rumen [51].

## Conclusion

The extract of *C*. *imbricata* demonstrated the lowest LD_50_ and LD_99_ values, making it the most effective wild desert plant extract tested for EHI. In the larval mortality test, *C*. *imbricata* was the second most effective extract, following *P. incanum*, which showed the highest efficacy against GIN larvae in cattle. This study is the first to evaluate desert plant extracts as a potential alternative for the control of GINs in cattle. Further research is needed to find the active ingredient and understand the mechanism of action that causes the mortality of eggs or larvae, and then design *in vivo* studies that demonstrate the effect of the active ingredients.

## List of abbreviations

EHI, egg hatching inhibition

GIN, Gastrointestinal nematode

gm, gram

h, hours

LD_50_, lethal dose 50

LD_99_, lethal dose 99

L_3_, third-stage larvae

mg, milligrams

min, minutes

ml, milliliter

µl, microliter.
